# Ultramini-percutaneous nephrolithotomy combined with flexible ureteroscopic lithotripsy for the treatment of complex renal calculi: a clinical study

**DOI:** 10.3389/fsurg.2025.1714640

**Published:** 2026-01-12

**Authors:** Fuyong Zhu, Chengjun Wang

**Affiliations:** Department of Urology, Qinghai Provincial People’s Hospital, Xining, China

**Keywords:** complex renal stones, flexible ureteroscopic lithotripsy, kidney injury, stone-free rate, stress response, ultramini-percutaneous nephrolithotomy

## Abstract

**Purpose:**

To compare the perioperative efficacy and safety of ultramini-percutaneous nephrolithotomy (ultramini-PCNL) combined with flexible ureteroscopic lithotripsy (FURL) vs. standard-channel PCNL in patients with complex renal calculi, with a focus on evaluating the impacts of the two procedures on postoperative oxidative stress responses, inflammatory factor levels, and renal hemodynamic parameters.

**Methods:**

A retrospective analysis was conducted on the clinical data of 135 patients with complex renal calculi admitted to our hospital from January 2023 to December 2024. According to different surgical approaches, the patients were divided into a study group (SG, *n* = 72) and a control group (CG, *n* = 63). The CG underwent standard-channel PCNL, while the SG received ultramini-PCNL combined with FURL. Surgical parameters, pain conditions, stone-free rate, oxidative stress markers, inflammatory factors, renal function, renal blood flow levels before and after surgery, as well as postoperative complications, were compared between the two groups.

**Results:**

Compared with the CG, perioperative blood volume, time to out of bed activity and hospital stay were shorter in the SG (*P* < 0.05); Postoperative pain scores were lower in the SG than in CG at 12 h, 24 h and 72 h (*P* < 0.05), postoperative stone-free rate was higher in the SG (*P* < 0.05); Postoperative Cor and MDA levels were lower while SOD levels were higher in SG (*P* < 0.05); Postoperative CRP, IL-6 and the complication rate were lower in SG than in CG (*P* < 0.05). Postoperatively, compared with the preoperative values, both groups showed slight fluctuations in serum urea and creatinine (Cr) levels; however, the differences were not statistically significant (*P* > 0.05). After surgery, the renal artery Vmax in the SG was significantly higher than that in the CG (*P* < 0.05). No significant differences were observed between the two groups regarding the RI index (*P* > 0.05).

**Conclusion:**

Ultramini-PCNL combined with FURL demonstrates superior efficacy in the treatment of complex renal calculi. This approach effectively improves stone-free rate, reduces stress responses, ameliorates inflammatory factor levels, and provides a high level of safety, supporting its broader clinical application**.**

## Introduction

Clinically, urinary tract stones are common in the department of urology and have the highest incidence rate in urological surgical department (1). Among them, upper urinary tract stones such as kidney stones are more common, which is very likely to cause complications such as urinary tract obstruction, urinary tract infection and even renal insufficiency, which affects the health and quality of life of patients (2, 3). Complex stones primarily refer to multiple, staghorn calculi. Most patients are accompanied by calyx neck stenosis, calyx dilatation or calyx anatomical abnormalities, which increases the difficulty of their treatment (4, 5). Complex stones are traditionally treated by open surgery, which could achieve better results but was more traumatic, leading to higher complication rate, hindering patients’ postoperative recovery (6). In recent years, with the continuous advancement of minimally invasive urological techniques, endoscopic combined intrarenal surgery (ECIRS) has become an important minimally invasive strategy for the treatment of complex renal calculi. It combines the advantages of percutaneous nephrolithotomy (PCNL) and flexible ureteroscopy, improving stone-free rates and reducing channel-related injuries. This study utilizes a sequential PCNL+ flexible ureteroscopic lithotripsy (FURL) combined strategy, which is an extension of the ECIRS concept (ECIRS-like sequential strategy). Furthermore, the approach further reduces the channel diameter (i.e., ultramini) to minimize renal parenchymal injury. Ultramini-percutaneous nephrolithotomy (ultramini-PCNL), based on traditional PCNL, further reduces the diameter of the surgical tract, thereby minimizing renal parenchymal injury, reducing intraoperative bleeding, and lowering the risk of postoperative complications (7). Meanwhile, FURL, owing to its excellent flexibility, can access hidden calyceal sites to effectively manage residual or difficult-to-reach stones, thus improving the stone-free rate (8). Although both procedures have distinct advantages, when used alone, they may not fully meet the therapeutic needs of complex renal calculi. Accordingly, in recent years, some researchers have attempted to combine ultramini-PCNL with FURL in order to increase the stone-free rate while simultaneously reducing tissue trauma and postoperative complications (9). However, current clinical studies on this combined approach remain relatively limited, and its true efficacy and safety still require further validation. Therefore, this study aimed to compare the clinical efficacy of ultramini-PCNL combined with FURL vs. standard-channel PCNL in the treatment of complex renal calculi, and to focus on the effects of the two procedures on postoperative oxidative stress responses, inflammatory factor levels, and renal hemodynamic changes, providing evidence for optimizing surgical strategies and perioperative management.

## Material and methods

### Baseline data

This study was a single-center, retrospective analysis that included complete clinical data of 126 patients with complex renal calculi who were admitted to our hospital between January 2023 and December 2024. All patients were diagnosed with complex renal calculi based on imaging examinations and clinical evaluation, and the lesions were unilateral. Inclusion criteria: (1) patients who were diagnosed with complex renal stones (9) by imaging and clinical diagnosis; (2) patients with unilateral stones and signed the informed consent. Exclusion criteria: (1) those with combined coagulation and immune dysfunction; (2) those with severe infections and malignant neoplastic disease; (3) those who had undergone urological surgery; (4) those with urethral malformations and contraindications to surgery. The study was approved by the ethics committee of Qinghai Provincial People's Hospital.

According to the type of surgical procedure, 63 patients who underwent standard-channel PCNL were assigned to the control group (CG), while 72 patients treated with super-mini PCNL combined with FURL were assigned to the study group (SG). There were no statistically significant differences between the two groups in baseline characteristics such as sex, age, stone location, or stone size (*P* > 0.05), indicating good comparability.

This study was conducted in strict accordance with the ethical principles of the Declaration of Helsinki (revised in 2013) and was approved by the Ethics Committee of our hospital. In the case screening stage of this study, the integrity of the data was strictly controlled. All included patients underwent routine testing for oxidative stress markers [cortisol (Cor), malondialdehyde (MDA), superoxide dismutase (SOD) and inflammatory factors [C-reactive protein (CRP), interleukin-6 (IL-6)]. To ensure the reliability of the analysis, this study only included patients with complete preoperative and postoperative data on relevant indicators. Therefore, there was no missing data or selective testing in this part.

### Methods

After insertion of an F5 ureteral catheter on the affected side, the patient was repositioned to the prone position, ensuring that the lumbar and dorsal regions were aligned on the same horizontal plane to facilitate puncture localization. Under ultrasound guidance, the target calyx containing the stone was identified. Normal saline was injected through the ureter to create artificial hydronephrosis, thereby expanding the collecting system and improving puncture visibility.

Following localization, percutaneous puncture of the target calyx was performed, and a zebra guidewire was inserted to establish the initial tract. In the CG, fascial dilators were used for sequential dilation from F8 to F24, after which a Peelway sheath was placed, and a Wolf F20.8 nephroscope was introduced for surgical manipulation. In the SG, under the same conditions, the tract was dilated only up to F12, followed by placement of a Peelway sheath and the use of a 7F ultra-mini nephroscope to perform super-mini PCNL.

In both groups, a 365 μm holmium laser fiber lithotripsy system was employed, with parameters set at an energy of 2.5–3.0 J and a frequency of 20–25 Hz. After fragmentation, larger stone fragments were removed using forceps. Intraoperative ultrasonography was used to verify the presence of residual stones. If residual fragments were detected, the ureteral catheter was removed and replaced with a flexible ureteroscope under guidewire guidance to inspect the calyces and hidden sites and to clear any remaining calculi.

At the end of the procedure, all patients were left with an indwelling F5 double-J stent and a nephrostomy tube to ensure unobstructed drainage and to reduce intrarenal pressure. Postoperatively, the nephrostomy tube was routinely removed approximately 1 week later, while the double-J stent was removed cystoscopically 4 weeks after surgery.

### Measurement

The surgical conditions, such as intraoperative bleeding, operative time, postoperative out of bed activity and hospitalization time, and decrease in hemoglobin (Hb) were compared.

The pain level of two groups was assessed and compared, and the pain level of the patients at 12 h, 24 h and 72 h postoperatively was analyzed using visual pain simulation scale, ranging 0–10 points, and the score was directly proportional to their pain level.

Stone removal and residuals were compared between two groups. The postoperative stone-free rate was evaluated using the current standard in urolithiasis treatment, defined as the absence of detectable residual stones or fragments (including clinically insignificant residual fragments) on postoperative imaging. Given the unique characteristics of complex renal calculi, this study still utilized the zero residual stone standards to assess stone-free rate. Clinically insignificant residual fragments (≤2 mm) were considered as residuals and were not included in the stone-free rate calculation.

The oxidative stress indicators of the two groups were detected. Fasting venous blood was drawn from the two groups before and 24 h after the operation, and the serum was collected by centrifugation. The serum Cor, MDA, and SOD indicators were detected using enzyme-linked immunosorbent assay.

The levels of inflammatory factors in two groups were compared, and their CRP levels were measured by automatic biochemical analyzer and the IL-6 levels were measured by enzyme-linked immunosorbent assay.

Renal function parameters: Peripheral venous blood samples were collected preoperatively and at 24 h postoperatively in both groups to measure blood urea nitrogen and serum creatinine (Scr) levels.

Renal hemodynamic parameters: According to the Chinese Guidelines for Diagnosis and Treatment of Urological Diseases (Urolithiasis Section) and the European Association of Urology (EAU) Urolithiasis Guidelines, postoperative imaging assessment of renal function and hemodynamic changes is recommended for patients undergoing PCNL. Color Doppler ultrasound, as a non-invasive and repeatable examination method, can be used to measure renal artery peak systolic velocity (Vmax) and resistance index (RI) and reflect postoperative renal parenchymal perfusion and potential damage. Therefore, Doppler ultrasound was implemented as a routine postoperative monitoring procedure in this study, and all included cases had complete examination data. Color Doppler ultrasound was performed preoperatively and at 24 h postoperatively in both groups to assess changes in Vmax and RI.

The incidence of complications: Both groups of patients underwent postoperative monitoring for complications, including incision infections, renal injury, and urinary fistulas. All complications were diagnosed according to urological clinical practice guidelines. Incision infection: defined by postoperative signs of redness, swelling, tenderness, exudate, or positive bacterial cultures from the wound; renal injury: postoperative imaging (e.g., ultrasound or CT) showing renal parenchymal damage, hematoma formation, or bleeding signs, or clinical manifestations related to renal injury; urinary fistula: persistent urine leakage from the nephrostomy tube or puncture tract beyond the expected recovery time, with or without local irritative symptoms; sepsis: based on the Sepsis-3 criteria, defined as the presence of clear evidence of infection accompanied by an increase in SOFA score ≥2 points, or the presence of systemic inflammatory responses such as fever/chills, tachycardia, tachypnea, altered consciousness, or elevated lactate levels. All complications were independently assessed by two senior urologists. In case of disagreement, a third senior expert made the final decision to enhance consistency and accuracy.

### Statistical analysis

SPSS 19.0 was used for data analysis. Count data (%) was examined by *X*^2^ test; Measurement data ($\bar{x}\pm s$) was examined by *t*-test, with *P* < 0.05 being significant difference.

## Results

### Comparison of baseline data

There was no statistical difference between two groups in terms of gender, age, stone diameter and location of the stones (*P* > 0.05) (Table 1).

**Table 1:** Comparison of baseline data between two groups (*x* ± *s*).

Group	Number of cases	Sex (m/f, cases)	Age (years)	Stone diameter (cm)	Stone location (left kidney/right kidney, *N*)
Control group	63	42/21	48.5 ± 5.4	2.91 ± 0.75	37/26
Study group	72	45/27	48.8 ± 5.1	2.83 ± 0.81	38/34
*t*	/	0.225	0.332	0.593	0.482
*P*	/	0.614	0.741	0.555	0.488

### Comparison of surgical indicators

**Figure 1:**
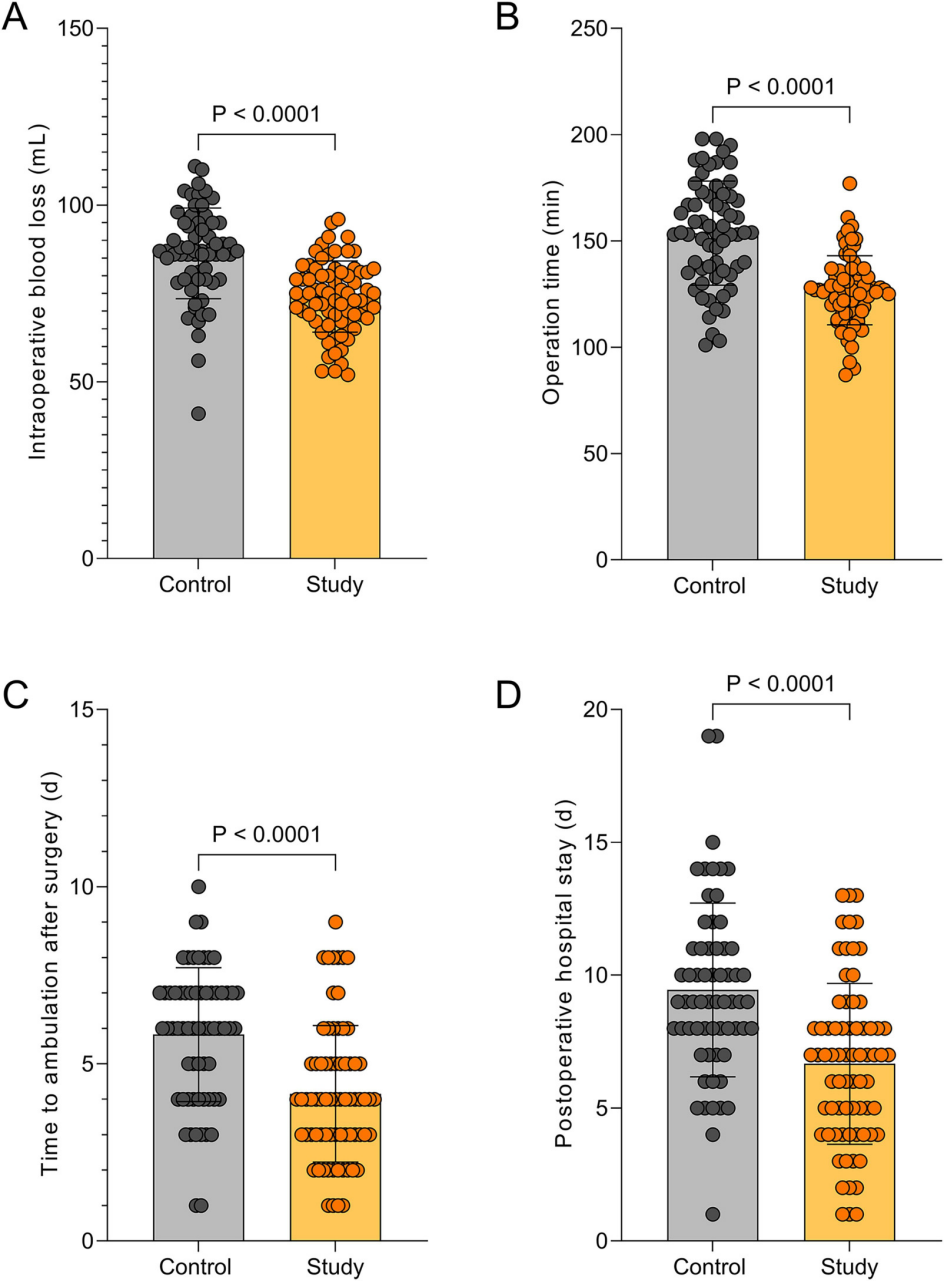
Comparison of surgical outcomes between the two groups ($\bar{x}\pm s$). **(A)** Intraoperative blood loss (mL); **(B)** Operative time (min); **(C)** Time to postoperative ambulation (day); **(D)** Postoperative hospital stay (day).

Intraoperative blood volume, postoperative out of bed activity and hospital stay were less or shorter in the SG than in CG (*P* < 0.05, Figure 1).

### Comparison of pain scores

**Figure 2:**
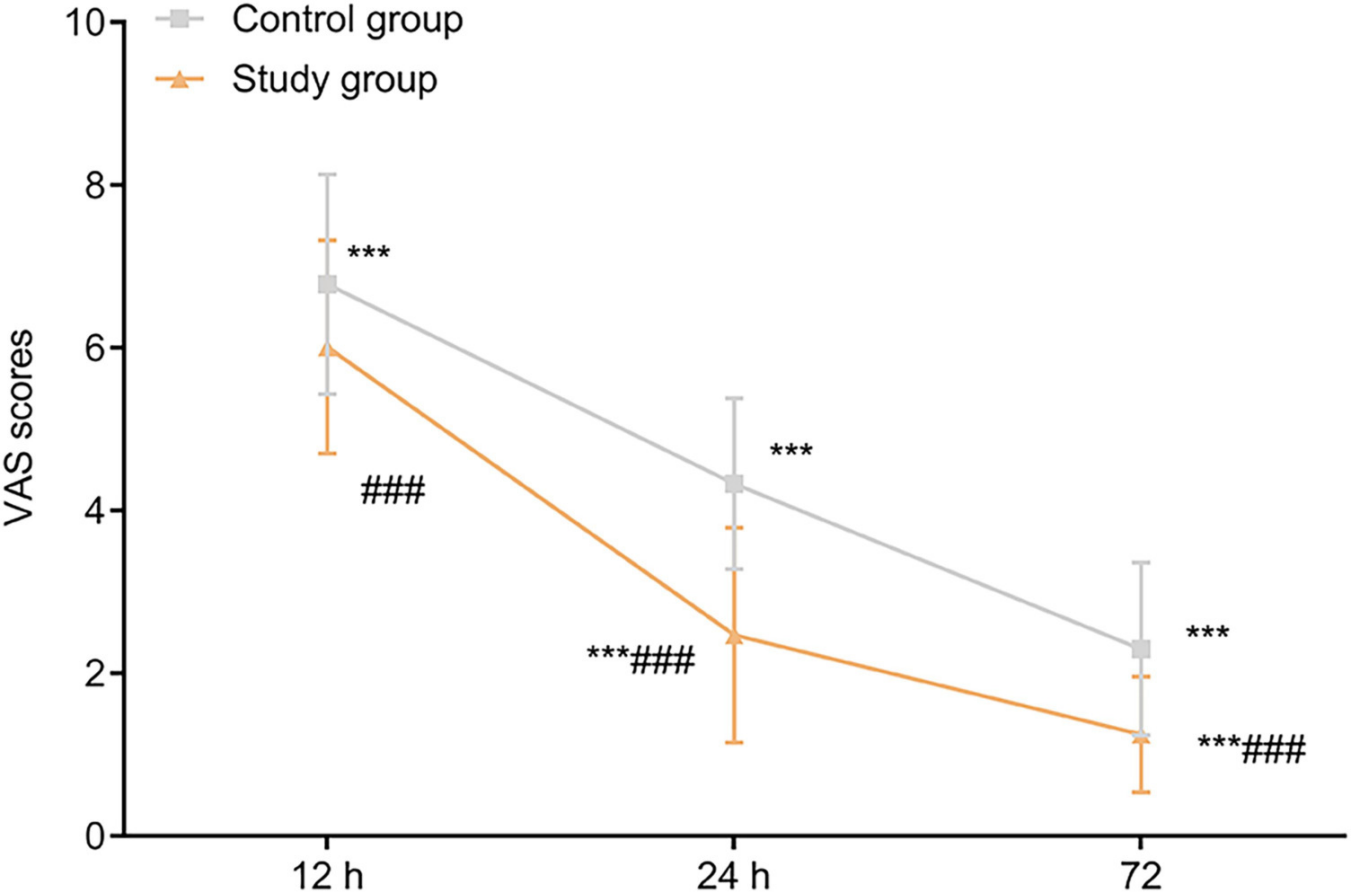
Comparison of pain scores between the two groups ($\bar{x}\pm s$, points).

The pain scores at 12 h, 24 h and 72 h postoperatively were lower in SG compared with CG (*P* < 0.05, Figure 2).

### Comparison of stone-free rate

**Figure 3:**
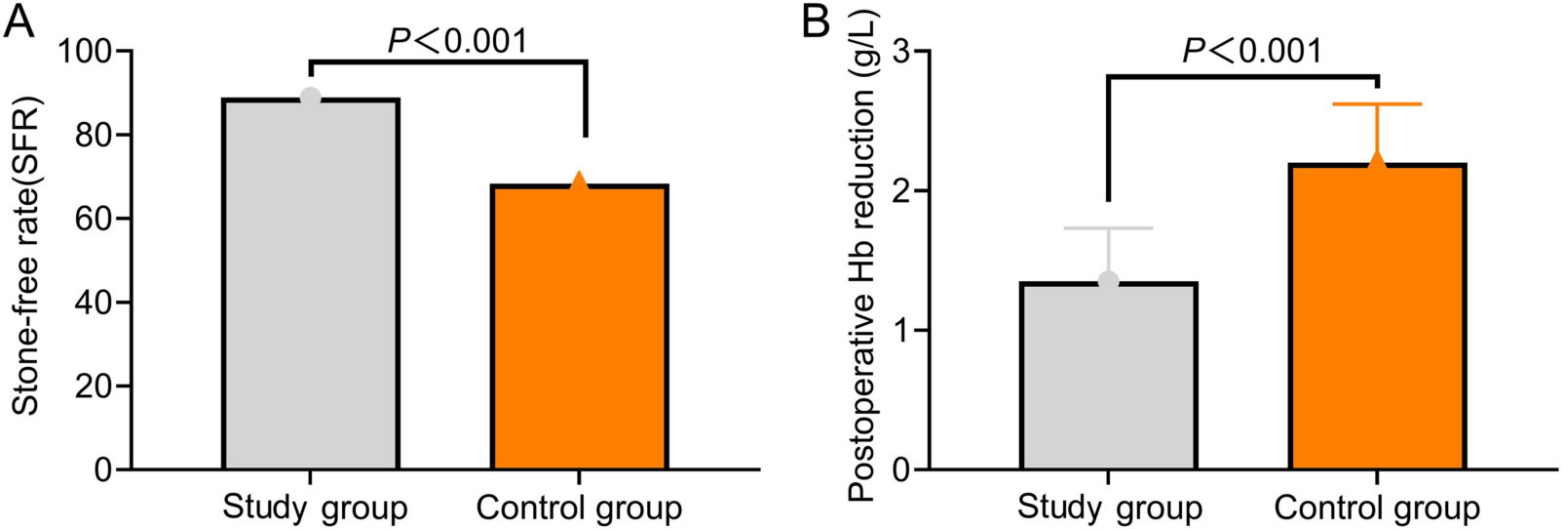
Comparison of stone-free rate and postoperative Hb reduction between the two groups (cases, %). **(A)** The stone-free rate in the study group was significantly higher than that in the control group; **(B)** The postoperative Hb reduction in the study group was significantly lower than that in the control group.

The stone-free rate in the SG was significantly higher than that in the CG, while the postoperative Hb reduction in the SG was significantly lower than that in the CG (*P* < 0.05, Figure 3).

### Comparison of oxidative stress response

**Figure 4:**
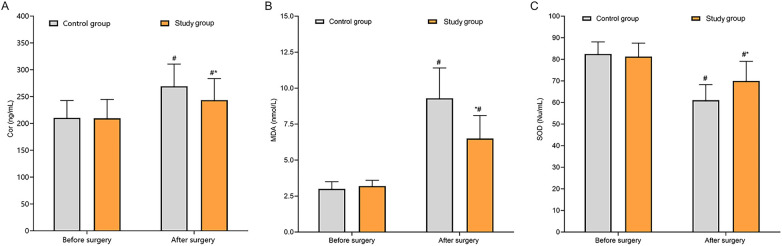
Comparison of oxidative stress indicators between the two groups. **(A)** The Cor level in the study group was significantly lower than that in the control group postoperatively; **(B)** The MDA level in the study group was significantly lower than that in the control group postoperatively; **(C)** The SOD level in the study group was significantly higher than that in the control group postoperatively. *P < 0.05 vs. control group.

Before treatment, the differences in Cor, MDA and SOD levels between two groups were not statistically significant (*P* > 0.05). After surgery, Cor and MDA levels were higher than those before treatment, while SOD levels were lower than those before treatment (*P* < 0.05). Compared with the CG, the SG had lower Cor and MDA levels and higher SOD levels (*P* < 0.05) (Figure 4).

### Comparison of inflammatory factor levels

**Figure 5:**
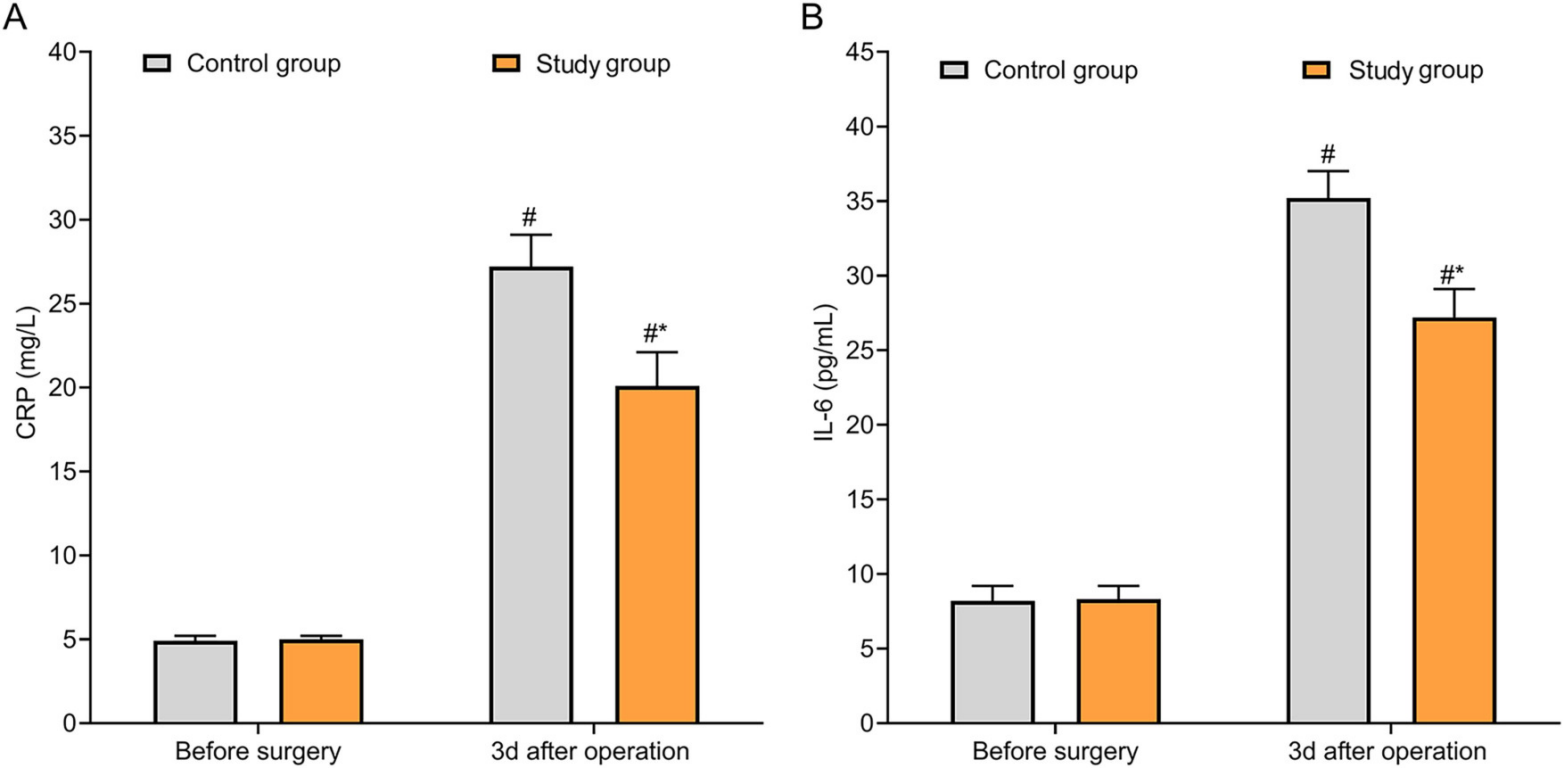
Comparison of inflammatory markers between the two groups. **(A)** The CRP level at 3 days postoperatively was significantly lower in the study group than in the control group; **(B)** The IL-6 level at 3 days postoperatively was significantly lower in the study group than in the control group. *P < 0.05 vs. control group.

Before treatment, the differences in CRP and IL-6 levels between two groups were not statistically significant (*P* > 0.05). The levels of CRP and IL-6 were higher in both groups 3 d after surgery than those before treatment (*P* < 0.05). CRP and IL-6 levels were lower in SG compared with those in CG 3 d postoperatively (*P* < 0.05, Figure 5).

### Changes in renal function and renal hemodynamic parameters between the two groups

Preoperatively, there was no significant difference in Urea and Cr levels between the two groups (*P* > 0.05), indicating comparability. Postoperatively, compared with the preoperative values, Urea and Cr levels in both groups showed slight fluctuations, but the differences were not statistically significant (*P* > 0.05), suggesting that renal function did not change significantly before and after surgery. Intergroup comparisons showed no significant differences in postoperative Urea and Cr levels between the two groups (*P* > 0.05), indicating that the interventions in this study had no obvious effect on renal function. Preoperatively, there was no significant difference in renal blood flow parameters between the two groups (*P* > 0.05). Postoperatively, the renal arterial Vmax in the observation group was significantly higher than that in the CG (*P* < 0.05). No significant difference was observed in RI between the two groups (*P* > 0.05, Table 2).

**Table 2:** Comparison of renal function and renal blood flow parameters between the two groups.

Group	Cases	Urea (mmol/L)		Cr (μmol/L)		Vmax(cm/s)		RI	
		Preoperative	Postoperative	Preoperative	Postoperative	Preoperative	Postoperative	Preoperative	Postoperative
Control group	63	3.17 ± 0.74	3.66 ± 0.80	63.71 ± 6.77	62.94 ± 6.88	70.29 ± 6.31	68.21 ± 5.24	0.64 ± 0.08	0.62 ± 0.07
Study group	72	3.24 ± 0.69	3.57 ± 0.62	64.16 ± 6.92	63.17 ± 6.51	69.75 ± 6.53	63.26 ± 6.09	0.65 ± 0.12	0.63 ± 0.11

### Comparison of complications between the two groups

**Figure 6:**
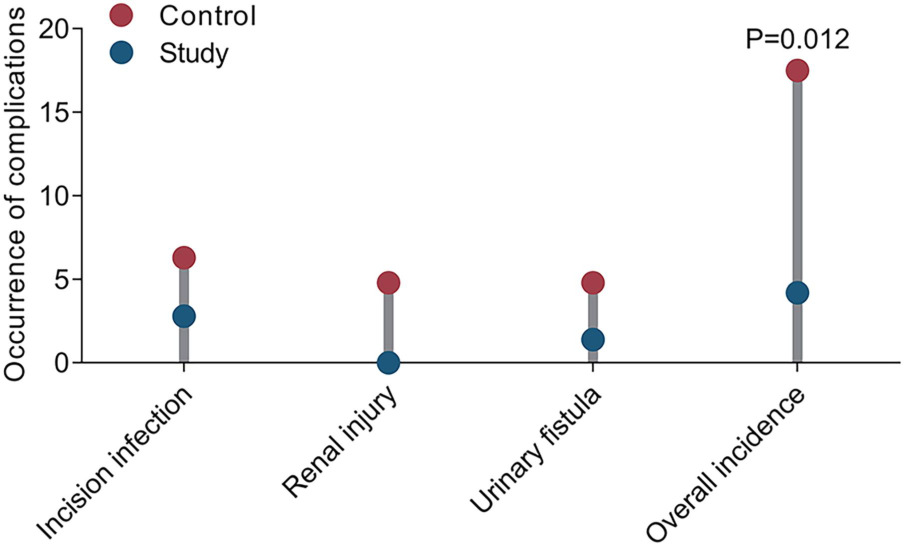
Comparison of complications between the two groups (cases, %).

There was a significant difference in the overall incidence of postoperative complications between the two groups. In the CG, 11 patients experienced postoperative complications, including 4 cases of incision infection (6.3%), 3 cases of renal injury (4.8%), and 3 cases of urinary fistula (4.8%), with an overall incidence of 17.5% (11/63). In contrast, only 3 patients experienced complications in the SG, including 2 cases of incision infection (2.8%), 1 case of urinary fistula (1.4%), and no cases of renal injury, with an overall incidence of 4.2% (3/72). The *χ*^2^ test results showed a statistically significant difference in the overall incidence of complications between the two groups (*χ*^2^ = 6.388, *P* = 0.012). The individual incidence rates of renal injury and urinary fistula were lower in the SG, but the individual comparisons did not reach statistical significance. However, it is noteworthy that neither group experienced sepsis postoperatively, suggesting that both surgical approaches have good safety profiles regarding postoperative infectious complications (Figure 6; Table 3).

**Table 3:** Comparison of complications between the two groups (*n*, %).

Group	Cases	Incision infection	Renal injury	Urinary fistula	Overall incidence
Control group	63	4 (6.3)	3 (4.8)	3 (4.8)	11 (17.5)
Study group	72	2 (2.8)	0 (0.0)	1 (1.4)	3 (4.2)
*χ* ^2^	–	–	–	–	6.388
*P*	–	–	–	–	0.012

## Discussion

In patients with complex kidney stones, some have urinary tract infection, coupled with the shape, distribution and size of the stones, which increases the difficulty of clinical treatment and affects the postoperative recovery (10, 11). Complex kidney stones tend to stay locally for a relatively long time, which could lead to ureteral obstruction, pain and hydronephrosis, and the condition may progress to uremia in serious cases, causing serious threats to patients’ health and life safety (12, 13). Complex kidney stones were traditionally treated by open nephrolithotomy, which has a high stone-free rate. However, open nephrolithotomy is an invasive operation, thus easily causing different degrees of damage to the kidneys, limiting its clinical application (14, 15). Therefore, it is crucial to formulate a reasonable, effective and safe treatment option.

In recent years, along with the advancement of medical technology and the gradual development of minimally invasive lumpectomy, new options for the treatment of patients with complex renal stones have gradually emerged (16, 17). For example, FURL and percutaneous nephrolithotripsy are minimally invasive and have fewer postoperative complications, promoting postoperative recovery (18, 19). However, complex renal stones often involve the renal calyces, renal pelvis, and ureteropelvic junction; due to their wide distribution and complex morphology, surgical management remains challenging (20, 21). Traditional PCNL commonly employs F24–26 standard tracts, which may cause substantial renal parenchymal injury and increase the risk of postoperative complications. While reducing the tract diameter can minimize renal tissue damage, it may also increase procedural difficulty (22). At present, no consensus exists regarding the optimal tract size for PCNL. Therefore, the combined application of PCNL and flexible ureteroscopy has been internationally classified as ECIRS and is recognized as an important minimally invasive approach for the management of omplex renal calculi.

In this context, the present study aimed to investigate the feasibility of using ultra-mini percutaneous tracts in PCNL combined with FURL and to further evaluate their impact on perioperative parameters, renal function, and complication rates, with the goal of providing a reference for optimizing clinical surgical strategies. Our findings demonstrated that, compared with the CG, the SG exhibited reduced intraoperative blood loss, shorter operative time, earlier postoperative mobilization, and shorter hospital stay. Additionally, postoperative pain scores at 12, 24, and 72 h were lower in the SG, and stone-free rate was higher in the SG. These results indicate that ultramini-PCNL provides favorable clinical outcomes for patients with complex renal stones, effectively alleviating postoperative pain, improving stone-free rate, and accelerating recovery.

The underlying mechanism may be that, compared with traditional F24–26 standard tracts, the smaller diameter of ultra-mini tracts significantly reduces damage to the renal parenchyma and vasculature, thereby decreasing intraoperative bleeding and postoperative complication risk. Moreover, reduced surgical trauma attenuates inflammatory responses, alleviates postoperative pain, and promotes earlier mobilization and shorter hospitalization. The combined use of ultra-mini tracts and flexible ureteroscopy allows complementary advantages, ensuring efficient clearance of large stones while managing hidden and small fragments, thereby enhancing stone-free rates and minimizing residual stones. Importantly, this approach minimally interferes with renal function, contributing to postoperative renal protection. Therefore, ultramini-PCNL combined with FURL not only improves the completeness of stone removal but also demonstrates higher clinical value in terms of safety and accelerated recovery.

Due to the large volume and extensive distribution of calculi in patients with complex renal calculi, calyceal dilatation, calyceal neck stenosis, and compression of the renal parenchyma are frequently present, thereby leading to changes in local renal hemodynamics. Long-term stimulation by renal calculi can cause impaired renal microcirculation, reduced perfusion, and local ischemia-reperfusion injury, placing the kidney in a state of persistent hypoperfusion and high stress. This is characterized by increased reactive oxygen species generation and decreased antioxidant defense capacity. These changes are not only associated with the chronic stimulation and inflammatory response caused by the stones themselves, but also closely related to the insufficient local perfusion resulting from calyceal obstruction. Persistent oxidative stress can further exacerbate renal parenchymal injury and worsen local hemodynamics, creating a vicious cycle that increases the surgical risk and the difficulty of postoperative recovery in patients with complex renal calculi. The results of this study indicated that postoperatively, both groups showed increased Cor and MDA levels and decreased SOD levels compared with preoperative values, indicating that surgical stress enhances oxidative stress responses. However, the SG exhibited smaller postoperative increases in Cor and MDA and a milder decrease in SOD compared with the CG, suggesting that ultramini-PCNL combined with FURL confers greater advantages in reducing oxidative damage and improving antioxidant status. Inflammatory marker analysis supported this conclusion: on postoperative day 3, both groups had elevated CRP and IL-6 levels compared with preoperative values, but the increases were significantly smaller in the SG, indicating that this surgical approach effectively suppresses excessive inflammatory factor release and mitigates inflammatory responses. Thus, the use of a less invasive surgical tract not only reduces tissue damage during the procedure but also positively impacts postoperative oxidative stress and inflammation control.

Regarding renal function and renal hemodynamics, preoperative urea and creatinine levels were comparable between the two groups. Postoperatively, although minor fluctuations were observed, there was no statistically significant difference, indicating that neither traditional nor ultramini-PCNL combined with FURL had a significant short-term impact on renal function. This aligns with previous reports showing that careful control of operative time and procedural extent can minimize damage to glomerular filtration (23). Notably, in terms of renal hemodynamics, preoperative parameters were similar between groups; postoperatively, peak renal artery velocity (Vmax) was significantly higher in the SG, while resistive index (RI) differences were not significant, suggesting that ultra-mini tracts better preserve renal blood flow and vascular patency. Typically, renal blood flow is low-resistance, and increased vascular resistance can reduce flow velocity, impairing renal perfusion. Previous studies have highlighted the close relationship between interlobar renal artery hemodynamics and renal function (24). During PCNL, tract dilation and mechanical manipulation can compress or damage adjacent tissue and vessels, potentially injuring interlobar or arcuate arteries and leading to perirenal hematoma, thereby affecting intrarenal blood flow (25, 26). Our results indicate that the smaller diameter of ultra-mini tracts minimizes interference with renal parenchyma and vasculature, reduces local vascular resistance, and lowers the risk of hemodynamic abnormalities and postoperative renal impairment. Consistent with surgical stress indicators, postoperative increases in Cor were lower in the SG, further supporting that this approach mitigates surgical stress and promotes recovery. Complication rates did not differ significantly between groups, indicating that ultramini-PCNL combined with FURL achieves effective stone-free rate while maintaining a favorable safety profile.

In conclusion, for patients with complex renal stones, ultramini-PCNL combined with FURL provides superior outcomes, effectively enhances stone-free rate, reduces systemic stress responses, improves inflammatory marker profiles, and demonstrates high safety, supporting its broader clinical application. Although the findings of this study are consistent with multiple previous studies, its evidence level is relatively limited due to the retrospective, single-center design. Therefore, the persuasiveness and originality of the study need to be further strengthened. However, in this study, oxidative stress markers and renal hemodynamic parameters were simultaneously included in the analysis, which showed greater physiological depth than previous research that mainly focused on surgical indicators or stone-free rates. The results of this study further confirm the advantages of ultramini-PCNL combined with FURL in reducing renal tissue injury, mitigating postoperative stress responses, and maintaining renal hemodynamic stability. These findings provide multidimensional evidence supporting the mechanistic value of this procedure. Despite the rapid adoption of advanced techniques such as supine PCNL, negative pressure suction systems, and FANS in some large medical centers in recent years, the their widespread adoption still varies significantly across different regions and among healthcare institutions of various levels. For medical institutions in primary-level or underdeveloped regions where advanced technologies may not be fully available, the combination of ultramini-PCNL and FURL remains highly practical and clinically feasible due to its low equipment dependency, high technical accessibility, and minimal invasiveness. The findings of this study also provide evidence-based support for such healthcare settings and have significant implications for broader application. Overall, this study demonstrates the value of the procedure from multiple perspectives, including surgical efficacy, physiological indicators, and clinical applicability. However, to further enhance the reliability and scientific rigor of these findings, future research should focus on large-scale, multicenter prospective studies to strengthen the level of evidence, validate the advantages of the procedure, and comprehensively evaluate its long-term efficacy under varying healthcare resource conditions.

## Data Availability

The original contributions presented in the study are included in the article/Supplementary Material, further inquiries can be directed to the corresponding author.
